# Unveiling the effects of tautomerism, pH, and solvation on the UV/Vis spectrum of sulfapyridine: a joint theoretical and experimental study

**DOI:** 10.1007/s00894-026-06933-y

**Published:** 2026-09-12

**Authors:** José L. F. Santos, Lorena C. P. Sales, Fernanda L. Souza, Gabriel L. C. de Souza, Adrian E. Roitberg, Roberto L. A. Haiduke

**Affiliations:** 1 Departamento de Química e Física Molecular, Instituto de Química de São Carlos, Universidade de São Paulo, Av. Trab. São Carlense 400, São Carlos, 13566-590 São Paulo Brazil; 2 Department of Chemistry, University of Florida, Gainesville, FL 32611 USA; 3 Centro de Ciências da Natureza, Universidade Federal de São Carlos, Rod. Lauri Simões de Barros, km 12, Buri, 18290-000 São Paulo Brazil

**Keywords:** Sulfapyridine, Tautomerism, Hybrid solvation, UV/Vis spectra, Density functional theory

## Abstract

**Context:**

Sulfapyridine (SPY) is a sulfonamide frequently detected as contaminant in aquatic environments, largely due to incomplete metabolism and its extensive use in livestock production and other anthropogenic activities. The spectroscopic properties of SPY are strongly influenced by prototropic tautomerism, pH conditions, and solvation effects. Herein, a joint theoretical–experimental study investigates the ultraviolet/visible (UV/Vis) electronic absorption spectra of SPY and its sulfonimide tautomer (TAUT) across different protonation states. Energetic analyses using a hybrid cluster-continuum (HCC) solvation approach reveal that TAUT forms predominate in aqueous solution. Hybrid solvation significantly lowers the tautomerization barriers (from 37.1 to 10.4 kcal.mol$^{-1}$ for neutral and from 35.4 to 8.6 kcal.mol$^{-1}$ for protonated forms), highlighting the catalytic role of the solvent. Theoretical data reproduce the main features of the experimental UV/Vis spectra, reinforcing that TAUT forms dominate in solution and exhibit characteristic absorption bands around 300 nm. These findings demonstrate that tautomeric equilibria and hybrid solvation are factors that strongly influence the spectroscopic properties of SPY in water and should be considered in studies involving sulfonamides, including biological interactions and photodegradation pathways.

**Methods:**

Conformational analyses are carried out at the GFN2-xTB/GBSA level (CREST). Cluster structures from SOLVATOR, with posterior geometry optimizations and vibrational frequency calculations, are achieved at the GFN2-xTB/ALPB level in water (ORCA 6.0.1). DFT calculations are performed at the PBE-D3/cc-pVTZ/PCM level (Gaussian 09) for providing equilibrium geometries for the lowest-energy conformers. Improved single-point energies are retrieved from CAM-B3LYP-D3(BJ)/def2-QZVPP/SMD calculations, while TD-DFT data reached at the CAM-B3LYP/def2-TZVP/C-PCM level are employed for UV/Vis spectra simulation (ORCA 5.0.4).

**Supplementary Information:**

The online version contains supplementary material available at https://doi.org/10.1007/s00894-026-06933-y.

## Introduction

**Fig. 1:**
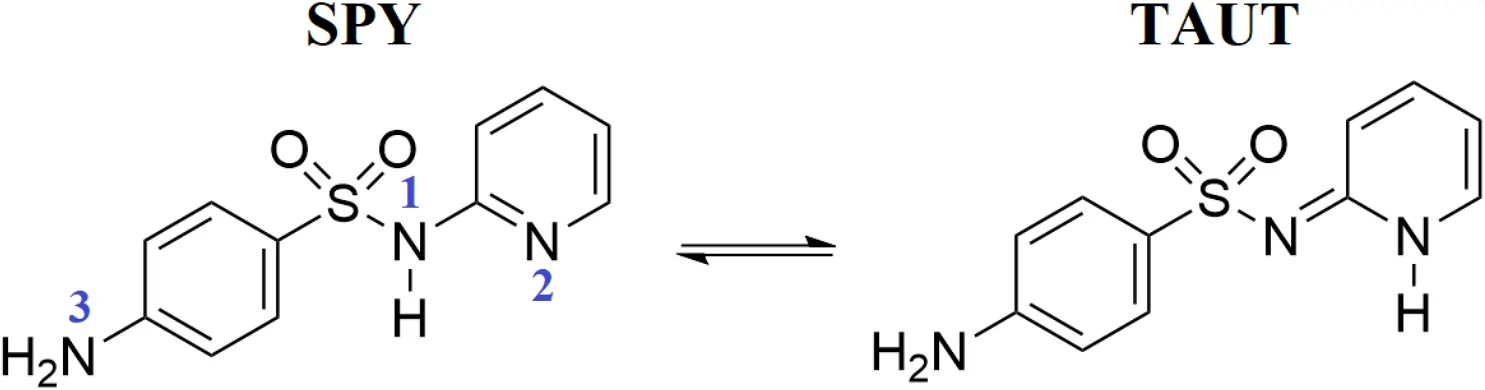
Chemical structures of sulfapyridine (SPY) and sulfonimide (TAUT) tautomeric forms

Tautomerism represents a form of isomerism involving the interconversion between distinct chemical structures — tautomers — through the transfer of an atom or functional group within the same molecular framework [1]. Among the different types of tautomerism, prototropic tautomerism is the most common one and involves the intra- or intermolecular proton transfer mechanism [2]. This process can occur in both ground and excited states and is commonly associated with keto-enol, amide-imide, amide-iminol, and lactim-lactone equilibria [3–5]. The tautomeric equilibrium has been studied since the nineteenth century and was critical in determining the structure of deoxyribose nucleic acid (DNA) [6]. Since then, many investigations have also explored the impact of tautomerism on drugs and its implications for mechanisms of action, delivery processes, metabolism, and toxicity profiles [2, 7, 8]. The excited-state proton transfer (ESIPT) mechanism is also related to many biological phenomena and, more recently, exhibits applications in fluorescent materials, chemical sensors, and optoelectronic devices [9, 10]. Analysis of drug databases shows that nearly 70% of all drug molecules exhibit tautomerization [11]. Consequently, disregarding tautomeric forms in experimental or computational studies can severely compromise the accuracy of the results and the mechanistic interpretations.

Sulfonamides are a class of antimicrobial compounds widely used in both human and veterinary medicine to treat bacterial infectious diseases [12]. Sulfonamide antibiotics (SAs) are also prone to tautomerism at ground and excited states [4, 13]. Sulfapyridine (SPY) is among the first SAs to be synthesized [14]. SPY has applications for treating infectious diseases in humans and, mainly, in agriculture and livestock activities [14, 15]. SPY may undergo structural variations due to the effects of pH [16] and tautomerism [17]. In the case of this compound, the tautomeric mechanism involves the proton transfer between two adjacent nitrogen atoms, leading to the formation of sulfonamide (SPY) and sulfonimide (TAUT) tautomers [18], as seen in Fig. 1. This process has been recognized since the 1950s, when studies on antibiotic development revealed that the coexistence of these tautomeric species in equilibrium could influence properties such as boiling points of methylated SPY and TAUT analogs and ultraviolet/visible (UV/Vis) spectra in alcoholic solution [18]. Computational studies in crystal structures of sulfonamide aggregates also showed a change in tautomeric equilibrium toward the sulfonimide tautomer due to the network of hydrogen bonds (HBs) [19]. Moreover, there are also some doubts regarding the structure of SPY in acidic conditions, with the nitrogen atom within the pyridine group identified as the preferred protonation site in the gas phase [17].

In addition to processes of biological importance, tautomerism can affect the environmental behavior of SAs and other chemical compounds [20, 21]. The low absorption and the excessive use of antibiotics, mainly in agriculture and animal husbandry, is responsible for the frequent detection of SAs in aquatic environments [22, 23]. Thereby, concerns arise on the remediation and consequences of water contamination to human health and environment [23, 24]. Since the tautomers can have different degradation routes, understanding the tautomeric equilibrium of SAs in aqueous solution can also render more effective photodegradation methodologies. Furthermore, the tautomeric equilibrium may be influenced by external conditions such as solvent, pH, and electric fields [25–28].

Unfortunately, most of experimental or theoretical studies on photodegradation of SAs do not include the effects of tautomeric forms in solution [29–32]. Tautomers are also neglected in some UV–Vis investigations of SPY in aqueous solution [16]. However, considering that these changes in molecular structure can affect the electron density distributions and, consequently, chemical properties of SPY and other SAs, the evaluation of the role of tautomers seems essential to improve the understanding of these decomposition routes.

Advanced experimental techniques are required for accurate measurements of tautomer concentrations in solutions. For example, nuclear magnetic resonance (NMR) [33], Fourier-transform infrared (FT-IR) spectroscopy [34], gas and liquid chromatography with mass spectroscopy (GC- and LC-MS) can be used [35, 36]. On the other hand, molecular modeling by computational chemistry can offer a valuable and less expensive tool to understand the behavior of tautomers in solution [37, 38]. Very accurate results can be obtained in gas-phase and implicit solvation in some cases. For instance, the use of high-level composite methods combined with implicit solvation models can provide accurate estimates of the energy differences between keto and enol salicylideneanilines tautomers [39, 40]. However, modeling reactions and UV/Vis spectra in protic solvents are still considered challenging, mainly because of problems that arise due to the inappropriate representation of hydrogen-bond interactions by implicit solvation models. In this context, the inclusion of explicit solvent molecules along with implicit solvation models — hybrid solvation — to account for these interactions on tautomeric mechanisms can improve the modeling reliability of these compounds [41–43]. Moreover, different tautomers may exhibit distinct absorption spectra due to variations in electronic distributions and molecular structures [18, 44], which can be used to identify and quantify their concentration in solution and mixtures at different pH conditions, providing valuable insights into the composition and behavior of the studied compounds.

In this work, we carried out a joint theoretical and experimental investigation about the effects of tautomerism, pH, and hybrid solvation on the energetics and ultraviolet/visible (UV/Vis) spectra of SPY in aqueous solutions. First, a conformational analysis was performed with the semi-empirical extended tight-binding (GFN2-xTB) method to probe for the lowest-energy structures under different protonation states in water. At this stage, solvation effects on relative Gibbs energies were included by adding up to 30 and 10 explicit water molecules to the neutral and the protonated/deprotonated forms, considering the hybrid cluster-continuum (HCC) approach, respectively. Next, Density Functional Theory (DFT) was used for evaluating the barrier heights for SPY tautomerization and the equilibrium between reactants and products. These reactions were performed with the lowest-energy protonated and neutral structures that allowed for tautomerism with up to two explicit waters. Time-dependent DFT (TD-DFT) was used to compute the UV/Vis data for the lowest-energy structures. The experimental UV/Vis absorption spectra were also obtained at different pH values (2, 7, and 11) and compared with the theoretical results predicted at each protonation state. Thus, we hope that these findings will contribute to the development of an accurate theoretical protocol for understanding the tautomeric equilibrium and absorption spectra of SAs and their tautomeric forms in natural aquatic environments as well as to providing a deeper knowledge regarding the effects of pH and hybrid solvation.

## Methodology

### Chemical and reagents

Sulfapyridine (purity > 99%) was supplied by Sigma-Aldrich (Merck KGaA, Darmstadt, Germany). $H_2SO_4$ and *NaOH* were purchased from Êxodo Científica (São Paulo, Brazil). Ultrapure water was obtained from a Milli-Q purification system (Millipore, Merck KGaA, Darmstadt, Germany).

### Chemical analyses

The electronic absorption spectrum of sulfapyridine was acquired using a UV–Vis spectrophotometer (UV-2600, Shimadzu) at pH values of 2, 7, and 11. Spectra were recorded in the wavelength range of 185–400 nm with a data interval of 0.01 nm and a slit width of 1 nm. The concentration of sulfapyridine was 5 $mg.L^{-1}$ at a temperature of 25 $^\circ$C. A quartz cuvette with a 10 mm optical path length was used.

### Computational methods

#### Determination of neutral, protonated, and deprotonated structures in aqueous solutions

As there are some doubts regarding the lowest-energy structures of SPY and TAUT forms in aqueous solutions at different pH conditions and considering the existence of distinct protonation sites in these molecules, the first step was to investigate these particular aspects. Initially, the structures are explored by using the screening tools available in the conformer–rotamer ensemble sampling tool (CREST) [45] package at the GFN2-xTB [46] level of theory, combined with the generalized Born surface area (GBSA) [47] implicit solvation model with water. Thus, this protocol provided the lowest-lying conformers without explicit solvent molecules. In this context, all the possibilities investigated in Ref. [17] for protonated SPY are considered.

Next, the solvated clusters are obtained by using the automatic placement tool of explicit solvent molecules known as SOLVATOR in the ORCA 6.0.1 [48, 49] package. At this stage, up to 30 and 10 explicit water molecules are arranged around the neutral and protonated/deprotonated solute, respectively, with DOCKER, a very accurate swarm intelligence algorithm that locates the minimum energy structures in the presence of each additional solvent molecule. The GFN2-xTB method and the analytical linearized Poisson–Boltzmann (ALPB) [47] implicit solvent model with water are used in this step. Geometry optimizations and vibrational frequency calculations are carried out starting from the explicitly solvated cluster structures by using GFN2-xTB and ALPB implicit solvation with water, constituting the xTB HCC approach for Gibbs energies obtained at 298.15 K and 1 atm. These calculations are also carried out by using the ORCA 6.0.1 package [48, 49], and the VERYTIGHTOPT convergence criterion is employed in all geometry determinations, ensuring tighter convergence of the potential energy surface in order to minimize the occurrence of low-energy imaginary vibrational frequencies.

#### Tautomeric mechanism

The geometries of the lowest-lying solute conformers found before are re-optimized using DFT, with the PBE exchange-correlation functional [50] plus D3 dispersion corrections [51, 52], and the cc-pVTZ basis set [53]. Here, the polarizable continuum model (PCM) [54] is considered for implicit solvation with water. In addition, potential energy surface scans are employed to locate proper starting structures for the transition states (TSs) along the tautomerization mechanism, which are refined in the sequence. Moreover, up to two explicit water molecules are added to account for explicit solvation effects in this reaction. The explicit water molecules are manually placed around the two adjacent nitrogen atoms (*N*1 and *N*2), bearing in mind the intermolecular proton transfer mechanism. Equilibrium geometries and harmonic vibrational frequencies of all these species are obtained at the same level of theory. The Cartesian coordinates can be found in the Supplementary Information A. Thermal corrections to Gibbs energies including the zero-point energy ($G_{thermal}$) are also obtained at 298.15 K and 1 atm. Intrinsic reaction coordinate (IRC) calculations were carried out to confirm that each TS uniquely connects the reactants with products [55, 56]. All these electronic structure computations are performed with the Gaussian 09 package [57].

In the sequence, single-point calculations are done with CAM-B3LYP [58] plus D3(BJ) [59, 60] empirical dispersion corrections along with def2-QZVPP [61] and def2/J [62] auxiliary basis sets in ORCA 5.0.4 [48, 63] to provide the best estimates of electronic energies of this study, $E_{el}(X)$. The solvation model based on density (SMD) [64] formalism is employed for implicit solvation with water, being considered as an improvement over common alternatives based on PCM. This combined methodology will be labeled here as CAM-B3LYP/QZ//PBE/TZ.

The final Gibbs energies of tautomerization, $\Delta G_{r}$, and activation, $\Delta G^{\ddagger }$, in aqueous solution are given by

1 $$\begin{aligned} \Delta G_{r} = G_{TAUT} - G_{SPY}, \end{aligned}$$

and

2 $$\begin{aligned} \Delta G^{\ddagger } = G_{TS} - G_{SPY}, \end{aligned}$$

where $G_{TS}$, $G_{TAUT}$, and $G_{SPY}$ are the Gibbs energies of the TS, TAUT, and SPY structures, with similar equations for protonated species. These quantities are given by

3 $$\begin{aligned} \ G_{X} = E_{el}(X) + G_{thermal}(X) + G_{css}(X) + G_{other}(X). \end{aligned}$$

where X=TS, *SPY*, or *TAUT*. Here, $G_{css}$ corresponds to a correction for Gibbs energies that refers to the standard state change from concentrations equivalent to 1 atm (used in Gaussian 09) to 1 $mol.L^{-1}$. In addition, $G_{other}$ represents the corrections to Gibbs energies beyond the rigid-rotor-harmonic-oscillator (RRHO) approach, that is, a quasi-harmonic correction to the entropy and enthalpy contributions of vibrational modes below 100 $cm^{-1}$ [65, 66]. $G_{css}$ and $G_{other}$ values were calculated with Goodvibes 5.0 [67].

The rate constants ($k_r$) [68, 69] for the SPY tautomerization mechanism can be easily estimated at room temperature (*T* = 298.15 K) by using

4 $$\begin{aligned} k_r = k_w \frac{k_B T}{h}exp \left( -\frac{\Delta G^{\ddagger }}{RT}\right) , \end{aligned}$$

where $k_B$, *h*, and *R* are the Boltzmann, Planck, and ideal gas constants, respectively. Moreover, $k_w$ is the Wigner’s factor [70, 71] for correcting quantum tunneling effects over rate constants,

5 $$\begin{aligned} k_w = 1+\frac{1}{24}\left( \frac{hc|\omega _i|}{k_BT}\right) ^2 , \end{aligned}$$

being *c* and $\omega _i$ the light speed and the imaginary frequency value for the transition state, respectively.

**Fig. 2:**
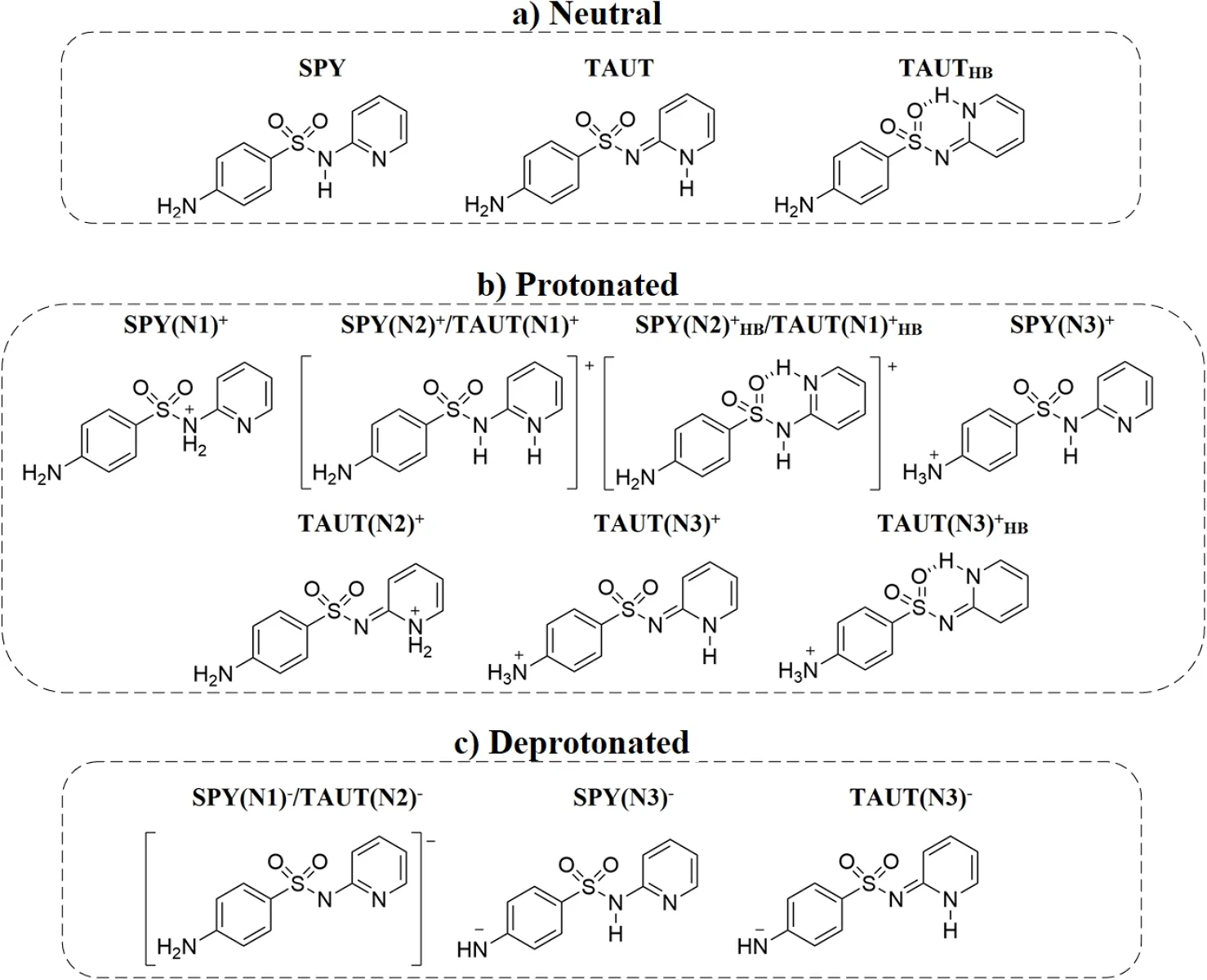
Chemical structures of all **a** neutral, **b** protonated, and **c** deprotonated tautomeric forms investigated in this work

**Fig. 3:**
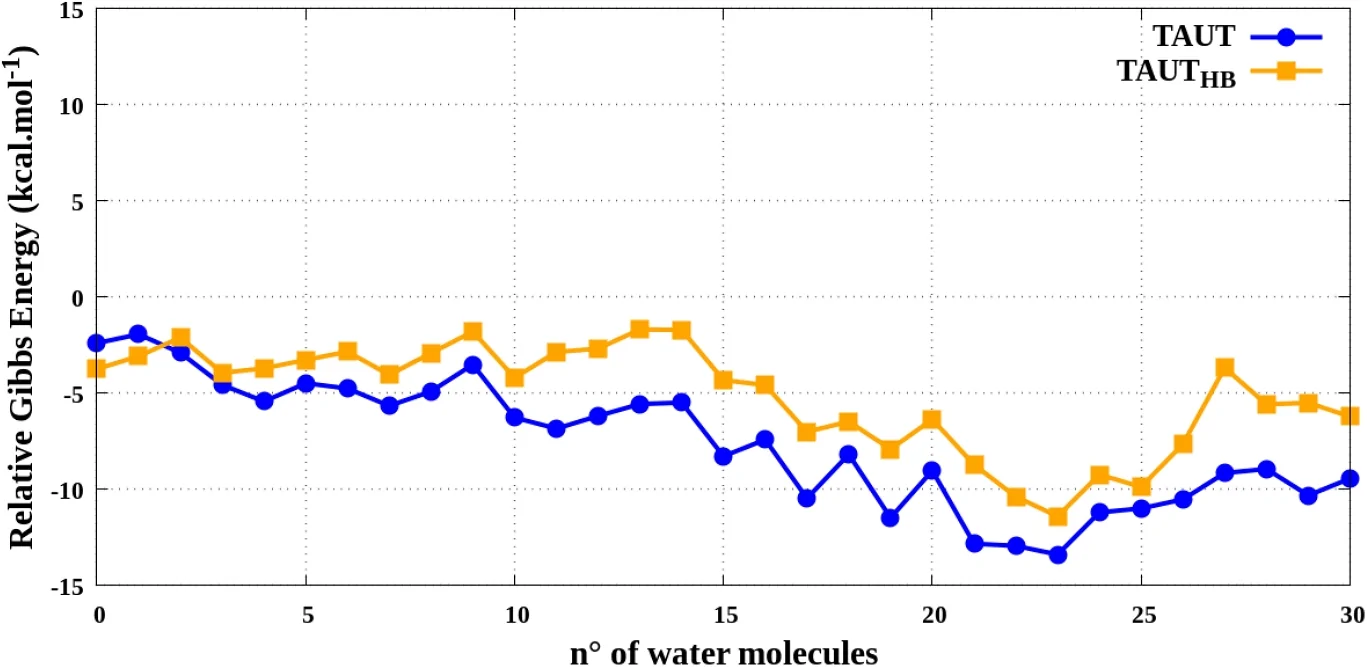
Gibbs energies of neutral forms [TAUT and TAUT$_{HB}$] relative to SPY obtained at xTB level along with implicit solvation and hybrid cluster-continuum (HCC) approaches with up to 30 explicit water molecules (in kcal.mol$^{-1}$)

**Fig. 4:**
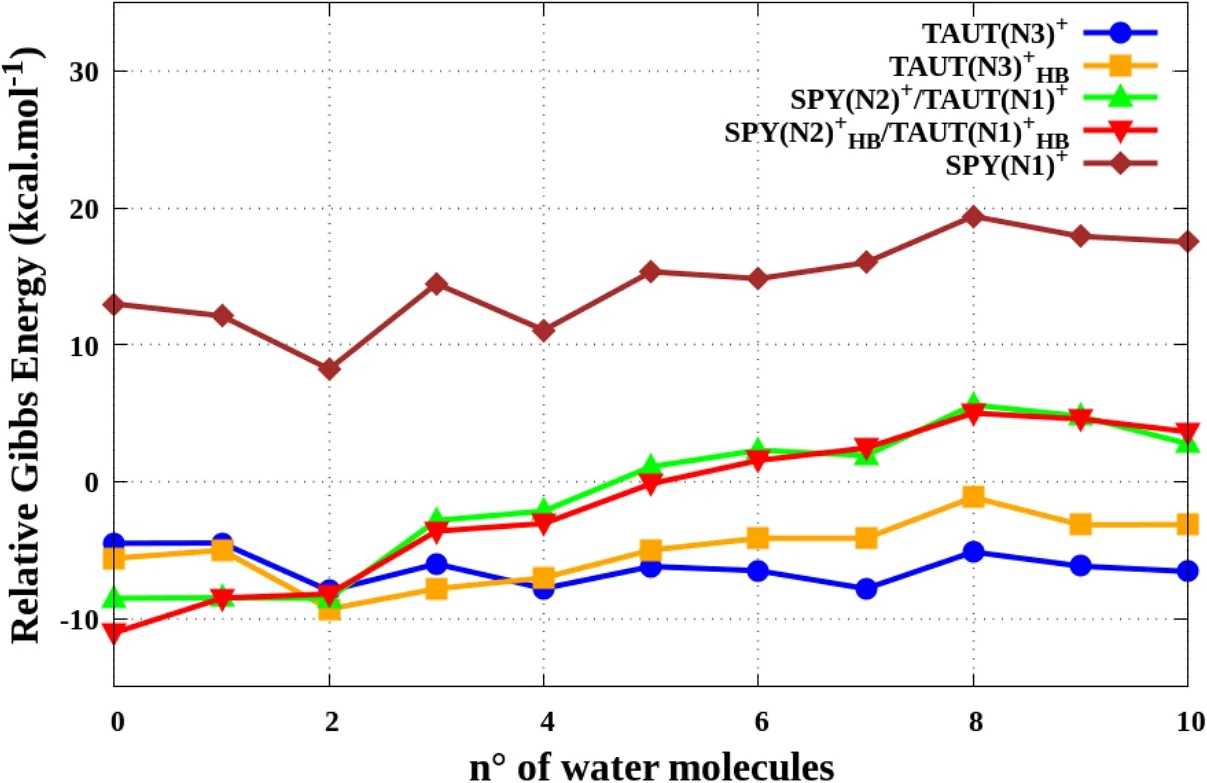
Gibbs energies of protonated forms [TAUT(N3)$^+$, TAUT$_{HB}$(N3)$^+$, SPY(N2)$^+$/TAUT(N1)$^+$, and SPY$_{HB}$(N2)$^+$/TAUT$_{HB}$(N1)$^+$] relative to SPY(N3)$^+$ obtained at xTB level along with implicit solvation and hybrid cluster-continuum (HCC) approaches with up to 10 explicit water molecules (in kcal.mol$^{-1}$)

**Fig. 5:**
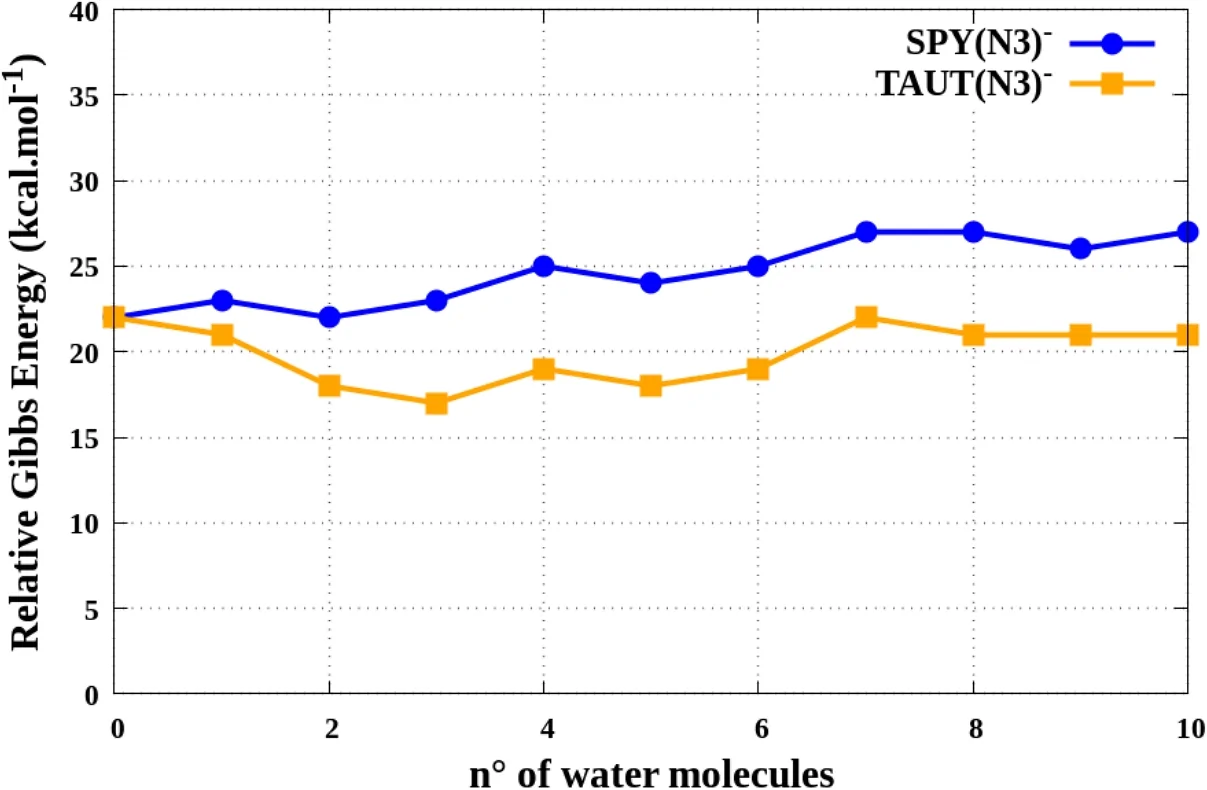
Gibbs energies of deprotonated forms [SPY(N3)$^-$ and TAUT(N3)$^-$] relative to SPY(N1)$^-$/TAUT(N2)$^-$ obtained at xTB level along with implicit solvation and hybrid cluster-continuum (HCC) approaches with 10 explicit water molecules (in kcal.mol$^{-1}$)

The tautomerization mechanism is a first-order process, but the inclusion of the explicit solvent molecules leads to complications. However, the tautomerism in terms of explicitly solvated clusters can still be treated as a first-order reaction. Therefore, the half-life time ($t_{1/2}$) is given by

6 $$\begin{aligned} t_{1/2}=\frac{ln2}{k_r}. \end{aligned}$$

#### Theoretical UV/Vis spectra

Taking the previously optimized geometries obtained at PBE-D3/cc-pVTZ level in water (PCM), TD-DFT is applied with CAM-B3LYP/def2-TZVP [58, 61] and def2/J [62] auxiliary basis set to obtain the UV/Vis spectra of all neutral, protonated, and deprotonated forms. These calculations are performed with implicit solvation, with the C-PCM model [72]. This combined methodology will be labeled here as CAM-B3LYP/TZ//PBE/TZ. The number of states requested is 30 for both singlet and triplet excitations. The TD-DFT calculations are performed in the ORCA 5.0.4 package [48, 63]. Natural transition orbitals (NTOs) are employed to provide a representation of the electronic transitions [73]. The molecular structures and NTOs are plotted using the ChimeraX 1.9 visualization package [74]. All spectra are convoluted with Lorentzian functions presenting a half-width of 11 nm in Gabedit 2.5.1 [75], which provides a better match compared to the experimental spectra.

**Fig. 6:**
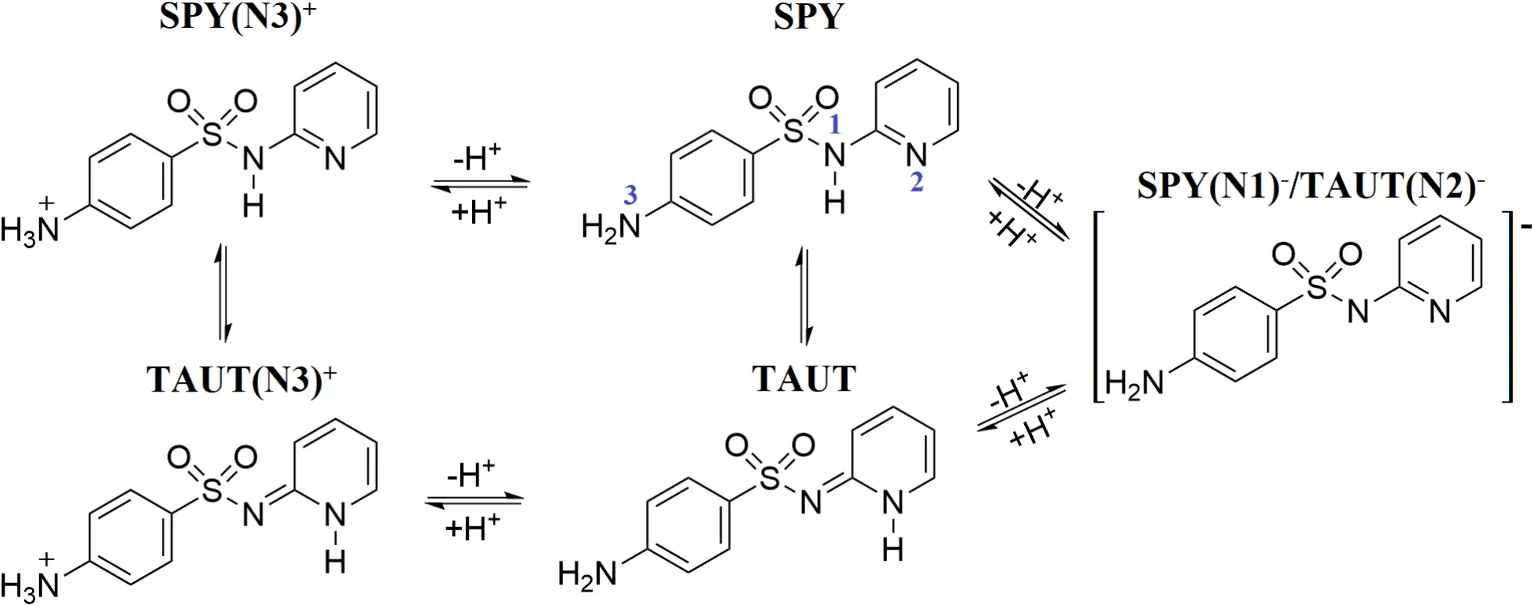
Main tautomerism equilibria of SPY under different protonation/deprotonation conditions in aqueous solution

## Results

### Conformational analysis of neutral, protonated, and deprotonated forms with implicit and hybrid cluster-continuum solvation

Figure 2 presents the chemical structures of the neutral, protonated, and deprotonated tautomeric forms investigated here. The label N*X* (*X* = 1, 2, and 3) indicates protonation or deprotonation in the corresponding nitrogen atoms (see Fig. 1). Tautomers featuring intramolecular hydrogen bonding are identified by the *HB* subscript. Distinct tautomeric pairs, such as SPY(N2)$^+$/TAUT(N1)$^+$ and SPY(N1)$^-$/TAUT(N2)$^-$, lead to equivalent protonated or deprotonated structures. Moreover, a protonated structure with a very high relative Gibbs energy with respect to SPY(N3)$^+$, identified using implicit solvation [TAUT(N2)$^+$, 33.6 kcal.mol$^{-1}$ at the xTB level], is no longer stable when explicit solvation is considered, as the proton is readily abstracted by solvent molecules. Therefore, the hybrid solvation analysis for TAUT(N2)$^+$ is not feasible.

**Fig. 7:**
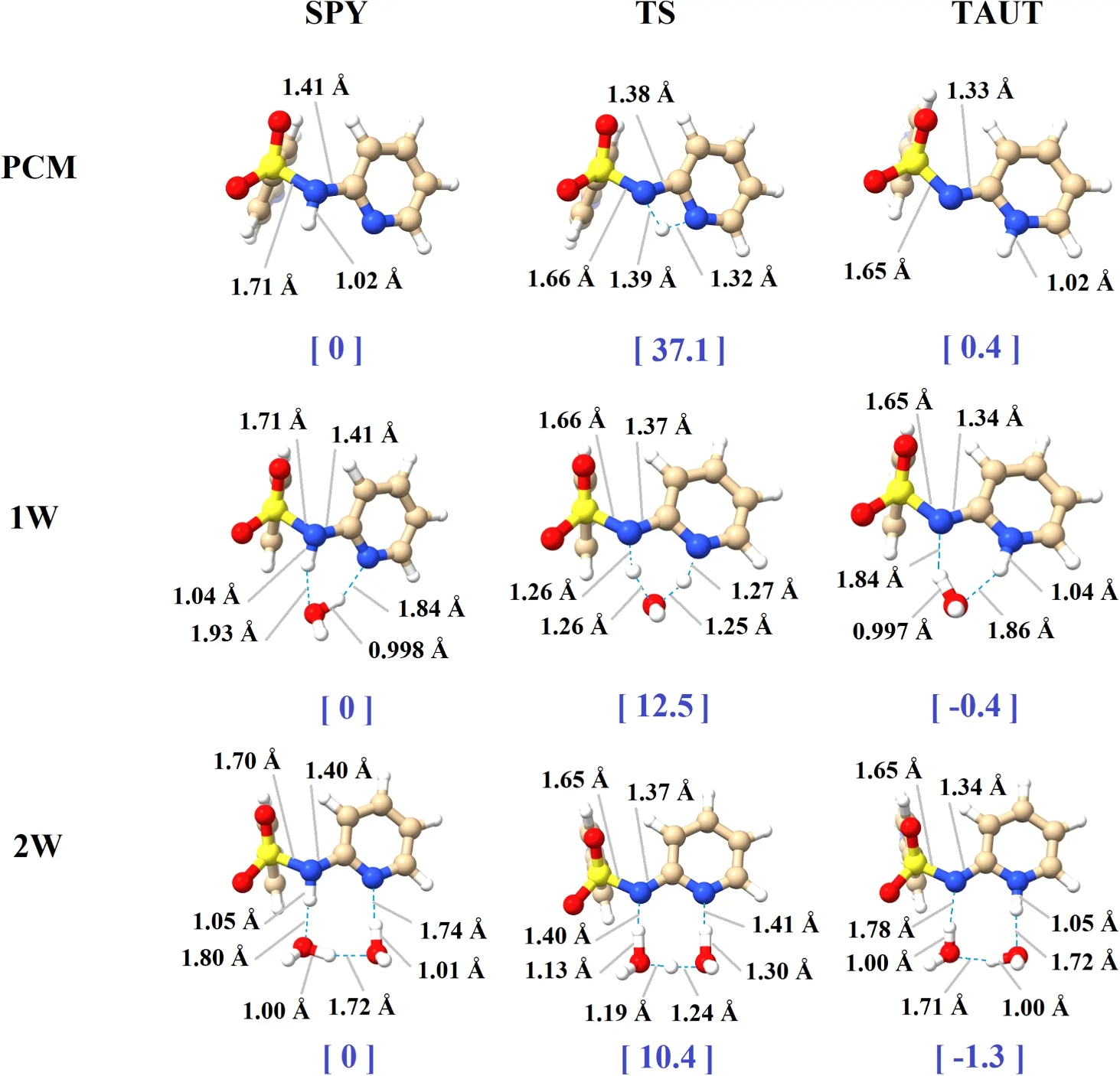
Equilibrium geometries obtained for the neutral SPY, TS, and TAUT forms at the PBE-D3/cc-pVTZ level in water with up to two explicit water molecules. Relative and activation Gibbs energies for the tautomerization reaction obtained at the CAM-B3LYP/QZ//PBE/TZ combined level are also displayed between square brackets (in kcal.mol$^{-1}$)

The relative Gibbs energies of the lowest-energy neutral, protonated, and deprotonated structures of SPY and TAUT found by the xTB method with implicit solvation (ALP) and within the HCC approach are presented in Figs. 3, 4, and 5, respectively. The results obtained with implicit solvation suggest that TAUT$_{HB}$ is the predominant form in aqueous solution for the neutral systems, whereas SPY$_{HB}$(N2)$^+$/TAUT$_{HB}$(N1)$^+$ corresponds to the predominant structure under implicit solvation for the protonated ones. The respective species without the intramolecular HB are slightly less favored by 1.3 and 2.5 kcal.mol$^{-1}$, respectively, for TAUT and SPY(N2)$^+$/TAUT(N1)$^+$. Similarly, [17] reported this protonation state as the dominant one in both the gas phase and methanol solution at the M06-2X/6-311++G(3df,3pd) level with IEF-PCM solvation. Finally, the results for the deprotonated forms show that SPY(N1)$^-$/TAUT(N2)$^-$ is by far the major species in aqueous solution (relative Gibbs energies of alternative structures are larger than 22 kcal.mol$^{-1}$).

**Fig. 8:**
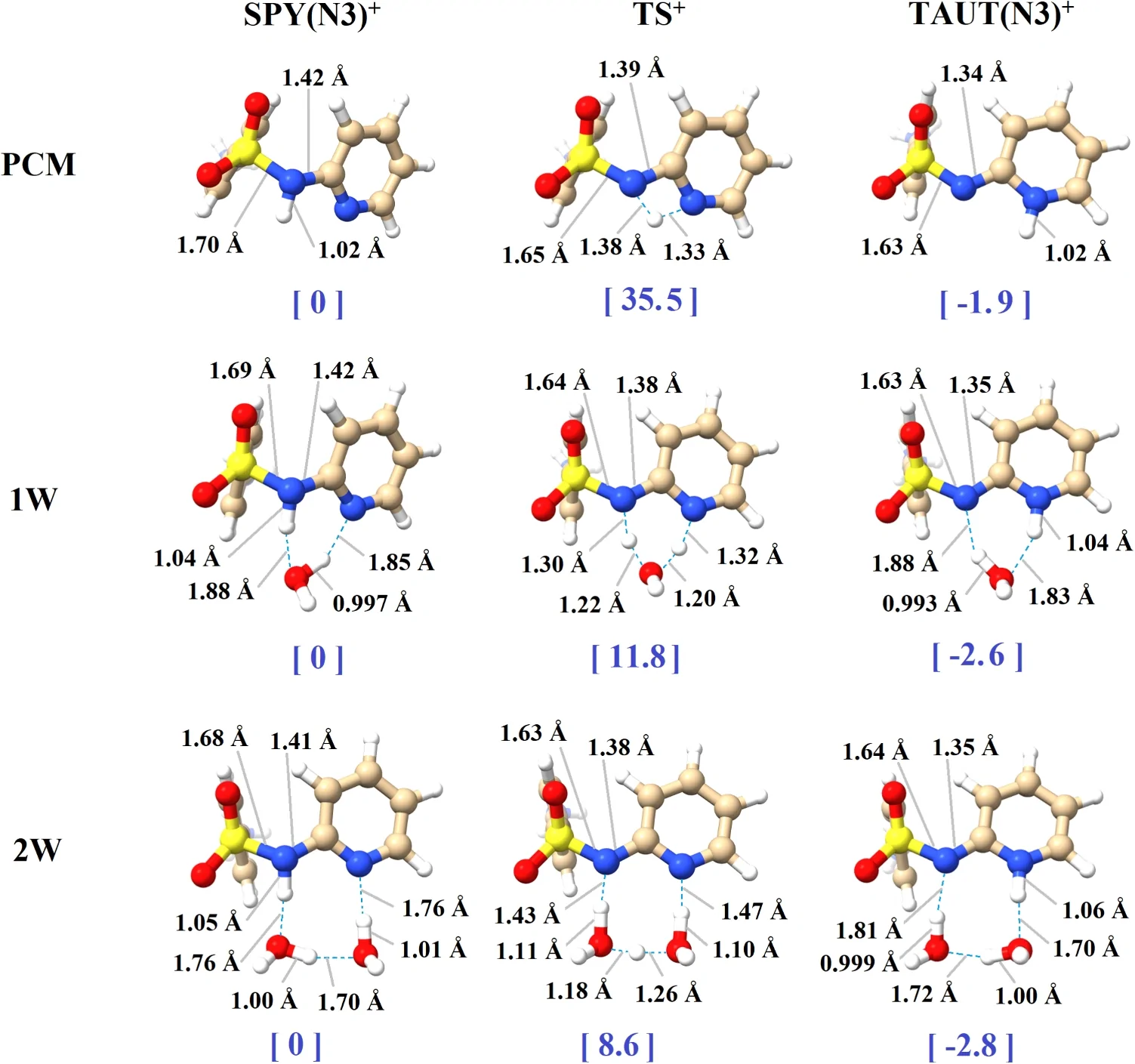
Equilibrium geometries obtained for the protonated SPY(N3)$^+$, TS$^+$, and TAUT(N3)$^+$ forms at the PBE-D3/cc-pVTZ level in water with up to two explicit water molecules. Relative and activation Gibbs energies for the tautomerization reaction obtained at the CAM-B3LYP/QZ//PBE/TZ combined level are also displayed between square brackets (in kcal.mol$^{-1}$)

Although these findings are consistent with previous studies based solely on implicit solvation models [17], hybrid solvation effects were also considered to better capture specific hydrogen-bonding interactions. Thus, using the protocol described within the HCC solvation framework, relative Gibbs energies were computed for explicitly solvated clusters containing up to 30 and 10 explicit water molecules for the neutral structures and protonated/deprotonated, respectively. For more details, the relative Gibbs energies given by xTB are available in Tables S1, S2, and S3 of the Supplementary Information B for the inclusion of each additional water molecule around neutral, protonated, and deprotonated solute forms.

Figures 3, 4, and 5 also show the relative Gibbs energies obtained with xTB inside the HCC solvation approach with water. The most remarkable changes can be seen in the neutral and protonated forms. Now, the inclusion of explicit solvation clearly indicates that TAUT becomes the majoritarian form in neutral medium (99.6%). Again, explicit solvation completely changes the equilibrium pattern of protonated structures in aqueous solution. Thus, while SPY$_{HB}$(N2)$^+$/ TAUT$_{HB}$(N1)$^+$ species was favored with implicit solvation only, the TAUT(N3)$^+$ form is now predominant within the HCC approach (99.7%). According to [76], the formation of strong solute–solvent interactions in protic environments can disrupt intramolecular hydrogen bonds, leading to stabilization of alternative conformations. Finally, the inclusion of explicit solvation does not change the behavior already observed with implicit solvation alone in the deprotonated forms. As one can see, the relative Gibbs energies of protonated/deprotonated structures show a nice convergence pattern with up to 10 explicit water molecules, whereas the convergence of the neutral forms is less well-behaved. This finding suggests that the electrostatic interactions between the charged species and the solvent seem to promote faster stabilization of the solvation shell.

In summary, the most relevant tautomeric equilibria under distinct protonated/deprotonated states can be understood as illustrated in Fig. 6, highlighting the main structures expected in aqueous solution. One should be careful with pure implicit solvation for protic solvents in conformational analysis, as the hydrogen-bonding interactions play an important role in the stabilization of these conformations.

### Barrier heights of tautomerization

After the conformational analysis, the most relevant structures obtained for each protonation state of SPY and TAUT (see Fig. 6) were re-optimized by using DFT at the PBE-D3/cc-pVTZ level of theory in water with PCM and used to investigate the tautomerism mechanism. The equilibrium structures of neutral and protonated forms of SPY and TAUT, along with the TS connecting them, are presented in Figs. 7 and 8. As mentioned before, up to two explicit water molecules are now included for hybrid solvation purposes. The relative energies and barrier heights of tautomerization provided by the CAM-B3LYP/QZ//PBE/TZ combined treatment in water are also shown in Figs. 7 and 8. Moreover, Table S4 of the Supplementary Information B also exhibits the calculated data for the tautomerization kinetics.

The interconversion between the SPY and TAUT forms proceeds through a barrier with $\Delta G^{\ddagger }$ of 37.1 kcal.mol$^{-1}$ considering only implicit solvation effects. Thus, the half-life time ($t_{1/2}$) related to this process would be around 1.6 million of years at room temperature, as seen in Table S4 of the Supplementary Information B. The analysis of the relative Gibbs energies of the tautomers also reveals that the SPY form is 0.4 kcal.mol$^{-1}$ lower than TAUT. However, this tautomerization barrier decreases drastically with hybrid solvation, going to 12.5 and 10.4 kcal.mol$^{-1}$ upon the addition of one and two explicit water molecules, respectively. In fact, this more advanced HCC treatment indicates that the interconversion from SPY to TAUT should occur almost instantaneously at room temperature, with $t_{1/2}$ values of 61.3 and 6.32 μs with the addition of one and two explicit water molecules, respectively. Furthermore, the TAUT form now exhibits a lower Gibbs energy than that of SPY by 1.3 kcal.mol$^{-1}$ with two explicit water molecules.

A similar pattern to the one observed for the tautomerization reaction of neutral SPY was also found for the protonated form, with the barrier decreasing from 35.5 kcal·mol$^{-1}$ under implicit solvation to 11.8 and 8.6 kcal·mol$^{-1}$, respectively, upon inclusion of one and two explicit water molecules. The best $t_{1/2}$ estimate is now 0.16 μs, which is even faster than the value obtained in the neutral medium. Interestingly, the formation of the protonated tautomeric form [TAUT(N3)$^+$] is already spontaneous with implicit solvation only. In addition, this species becomes even more favored with two water molecules, with a relative Gibbs energy reaching –2.8 kcal·mol$^{-1}$. Finally, tautomerism is not possible for SPY(N1)$^-$/TAUT(N2)$^-$ due to the deprotonation that occurs under basic conditions.

The reason for the drastic decrease in $\Delta G^{\ddagger }$ for the SPY tautomerism mechanism in both neutral and acidic media is clearly related to the proton transfer chain made possible by the explicit solvent molecules. The water molecules included act as both donors and acceptors of protons in the explicitly solvated cluster systems. The TS obtained with one and two explicit water molecules form six- and eight-membered rings, which probably result in structures with less tension. Then, the proton can be transferred in a concerted mechanism. The decrease in $\Delta G^{\ddagger }$ due to the catalytic effect of explicit water is frequently observed in organic and biological compounds, commonly resulting in barrier height decrements from 20 to 50 kcal.mol$^{-1}$ [77–79].

### Effects of pH and tautomeric forms on the UV/Vis absorption spectra

The electronic UV/Vis absorption spectra obtained at the CAM-B3LYP/TZ//PBE/TZ level in aqueous solution (C-PCM) for all the neutral, protonated, and deprotonated structures investigated here are presented in Figs. S1 – S3 of the Supplementary Information B. The results from the vertical excitation energy (VE), the generalized oscillator strength (GOS), and the NTOs obtained for the five lowest-lying singlet excited states of the most relevant species mentioned before are presented in Table S5 of the Supplementary Information B.

Generally, the computed electronic UV/Vis absorption spectra of SPY and its tautomeric forms exhibit significant differences in VE and GOS results and, consequently, in the overall spectral shapes. As noticed, the spectra of neutral structures displayed two bands/shoulders between 220 and 255 nm. However, the most distinctive feature in this case is the appearance of an additional low-energy band around 295 nm for TAUT and TAUT$_{HB}$, which can be used as a fingerprint of such tautomeric structures compared to SPY. Here, the intramolecular hydrogen bond induces a small redshift of the entire TAUT spectrum. For example, the fingerprint band of TAUT is centered at 292 nm, being displaced to 297 nm in TAUT$_{HB}$.

The protonated forms present more significant spectral variations due to the different protonation sites and tautomeric arrangements possible. For instance, SPY(N3)$^+$ presents only one peak around 235 nm, while TAUT(N3)$^+$ and TAUT$_{HB}$(N3)$^+$ also show a lower-energy band centered at 287 and 302 nm, respectively, highlighting the tautomeric fingerprint peak redshift. The TAUT(N1)$^+$/SPY(N2)$^+$ structure possesses a band around 261 nm with a shoulder nearby 280 nm. Again, the intramolecular hydrogen-bond interaction in TAUT$_{HB}$(N1)$^+$/SPY$_{HB}$(N2)$^+$ causes a redshift of the lowest-energy band, along with a GOS increase, which results in a prominent peak around 291 nm. For completeness, the spectra of the highest-energy protonated structures investigated, SPY(N1)$^+$ and TAUT(N2)$^+$, were also computed. Both SPY(N1)$^+$ and TAUT(N2)$^+$ exhibit their lowest-energy absorption bands at approximately 332 nm and 358 nm, respectively, together with two additional higher-energy transitions in the range between 238 and 302 nm.

Finally, in the case of deprotonated species, the highest-energy structures, SPY(N3)$^-$ and TAUT(N3)$^-$, present the most intense peak around 295 and 286 nm, respectively, with other small bands/shoulders at higher-energy regions, while the lowest-energy deprotonated form, SPY(N1)$^-$/TAUT(N2)$^-$, shows two almost merged peaks around 233–249 nm, with a less intense shoulder nearby 272 nm.

**Fig. 9:**
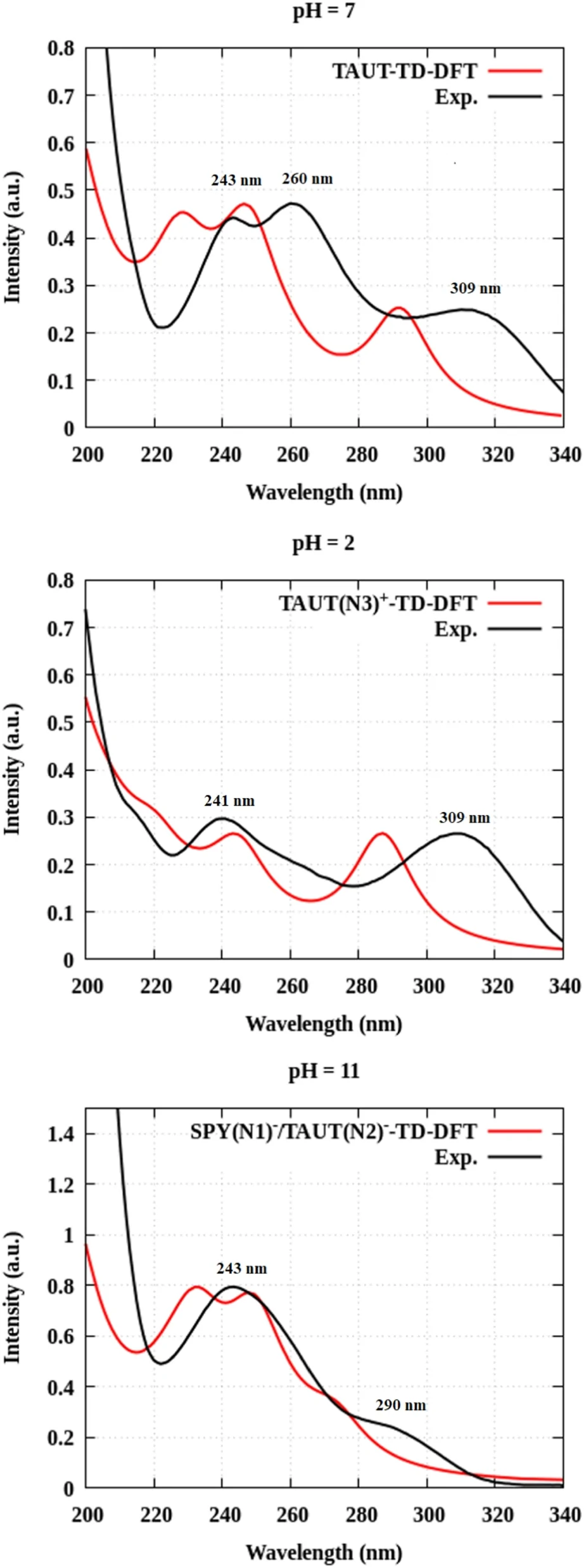
Electronic absorption UV/Vis spectra of TAUT and the structures predicted to occur in acidic [TAUT(N3)$^+$] and basic [SPY(N1)$^-$/TAUT(N2)$^-$] conditions determined at the CAM-B3LYP/TZ//PBE/TZ level with C-PCM (red lines). Experimental results are also plotted (black lines)

In the sequence, Fig. 9 illustrates the spectra of the majoritarian structures predicted in each protonation state along with the experimental absorption UV/Vis spectra acquired in aqueous solutions of SPY at acidic (pH = 2), neutral (pH = 7), and basic (pH = 11) conditions. The intensities of our theoretical spectra are normalized, taking the experiments as reference. Thus, in accordance with our discussions based on thermodynamic data, the experimental absorption spectra under neutral, acidic, and basic conditions are in nice agreement with our theoretical results obtained for TAUT, TAUT(N3)$^+$, and SPY(N1)$^-$/TAUT(N2)$^-$ forms at the CAM-B3LYP/TZ//PBE/TZ level with C-PCM, respectively. In addition, some redshift may also be expected by the hydrogen-bonding interactions with the solvent itself, which is not taken into account by our calculations.

This provides further evidence that tautomeric forms occur predominantly in aqueous solution and probably in other protic solvents. Experimentally, at neutral pH, three absorption peaks are observed at 243, 260, and 309 nm. At acidic pH, the band near 260 nm seems to be weakened, becoming a mere shoulder with respect to the peak at 241 nm. Under basic conditions, the lowest-energy band shows a pronounced intensity decrease and shifts to 290 nm, resulting in a shoulder in the spectra, while the other two bands probably merge together toward the single peak feature now visible at 243 nm. Hence, the TD-DFT spectra show similar features compared with the experimental data. These results are consistent with another previous experimental spectral study as well, although the authors tentatively interpreted the data obtained by supposing only the presence of SPY, SPY(N3)$^+$, and SPY(N1)$^-$, that is, without any tautomeric species [16].

Interestingly, the presence of tautomeric forms of SPY in neutral and acidic alcoholic media has been reported since 1951 [18]. In this context, absorption spectra obtained for purified methylated SPY and TAUT compounds, that is, structures preventing tautomerization by presenting a methyl group instead of the hydrogen atom usually transferred, were compared with the spectra of sulfapyridine solutions. Notably, the characteristic fingerprint peak associated with the imide tautomer is observed for the methylated TAUT form under both pH conditions, with peaks at 325 nm, in nice agreement with our results obtained in aqueous solution. These authors suggested that the spectra from sulfapyridine solutions refer to mixtures between the two tautomers. Thus, taking the absorption coefficient of methylated forms as reference, relative populations of the tautomeric species were estimated, suggesting values such as 30% and 70% for TAUT in neutral and acidic alcoholic media, respectively. This work also found evidence that the fingerprint band weakening can also be related to an equilibrium shift toward the amide form in less polar alcoholic media.

Once the predominant tautomeric forms of SPY in solution under different pH conditions are elucidated [TAUT, TAUT(N3)$^+$, and SPY(N1)$^-$/TAUT(N2)$^-$] and the electronic singlet states ($S_n$) related to each active transition are assigned [TAUT = $S_1$, $S_3$, and $S_5$; TAUT(N3)$^+$ = $S_1$, $S_2$, and $S_4$; SPY(N1)$^-$/TAUT(N2)$^-$ = $S_1$, $S_3$, and $S_4$], the main NTOs involved in these transitions can be seen in Figs. S4 – S6 in the Supplementary Information B. This analysis shows that such electronic transitions, which are involved in photodegradation processes of these contaminants, are explained as excitations between π orbitals extending across all systems. As one can see, the lowest-energy transition in all cases appears to be more localized at the pyridine ring moiety. Since this ring is affected directly by the intramolecular hydrogen bonding, a small redshift in the position of the fingerprint band due to this interaction seems to be not surprising.

## Conclusion

A joint theoretical and experimental investigation on the effects of tautomerism, pH, and hybrid solvation on the equilibrium and UV/Vis spectra of sulfapyridine tautomers in aqueous solution was carried out in this work. The xTB approach was used to obtain the relative Gibbs energies of the lowest-energy conformers with implicit solvation and with hybrid solvation by means of the HCC approach. DFT was employed to compute the barrier heights of tautomerization and the relative Gibbs energies with the inclusion of up to two explicit water molecules in the reaction mechanism, while TD-DFT was used to compute the excited-state properties.

The conformational analysis reveals the relevance of including hybrid solvation effects under the HCC approach, showing that the equilibria of the tautomeric forms in different pH conditions were altered. Thus, the TAUT, TAUT(N3)$^+$, and SPY(N1)$^-$/TAUT(N2)$^-$ structures are predicted as predominating in neutral, acidic, and basic conditions, respectively. The relative Gibbs energies obtained for these species with the HCC approach (explicitly solvated clusters with up to 30 and 10 explicit water molecules for neutral and protonated/deprotonated tautomers, respectively) allowed us to estimate populations of almost 100% for TAUT, TAUT(N3)$^+$, and SPY(N1)$^-$/TAUT(N2)$^-$ in neutral, acidic and basic aqueous solution, respectively.

The inclusion of hybrid solvation in the tautomerization mechanism is mandatory as well, since these barriers underwent a drastic decrease due to solvent effects. First, for the neutral system, the interconversion barrier decreases from 37.1 to 10.4 kcal·mol$^{-1}$ as going from implicit solvation to hybrid solvation with two explicit water molecules. In acidic medium, the tautomerization barrier decreases from 35.5 to 8.6 kcal·mol$^{-1}$ in the same context. Hence, the catalytic role of water molecules is crucial along this reaction. Furthermore, the presence of explicit solvent molecules favors the TAUT forms compared to the SPY structures along the chemical equilibria.

The analysis of the absorption spectra revealed the characteristic features of each molecule with different tautomeric forms and distinct protonation/deprotonation sites. TD-DFT spectra evidenced the presence of a fingerprint band ascribed to the tautomeric species around 287–302 nm in both neutral and acidic conditions. Thus, by comparing the theoretical with the experimental data, in accordance with the analysis of relative Gibbs energies, it was possible to show that the spectra measured in neutral (pH = 7) and acidic conditions (pH = 2) correspond to the TAUT and TAUT(N3)$^+$ forms, respectively. Based on the calculated interconversion barriers and the relative energies of the tautomeric species, this study concludes that the sulfonimide forms are generated in almost instantaneous reactions and predominate in aqueous solutions of sulfapyridine at room temperature. Therefore, the effect of tautomerism should be considered in future studies of sulfonamides in aqueous environments, as well as can significantly influence the light absorption properties and, consequently, photochemical and photophysical processes.

## Supplementary information

Supplementary Information A: Cartesian coordinates of equilibrium structures obtained with xTB and DFT methods (in Å).

Supplementary Information B: Tables with relative Gibbs energies for all neutral, protonated, and deprotonated structures obtained at the xTB level with HCC solvation. Table with thermodynamic and chemical kinetics data of tautomerization reactions obtained with DFT. Table with GOS and VE values along with the main NTO percentages for electronic transitions of predominant tautomers. Figures of computed spectra of all neutral, protonated, and deprotonated tautomeric forms. Figures illustrating the main NTOS involved in the transitions investigated.

## Supplementary Information

Below is the link to the electronic supplementary material.

Supplementary file 1 (zip 4107 KB)

## Data Availability

The data that support the findings of this study are available from the corresponding author upon reasonable request.
