# Supplementary material for: Unveiling the effects of tautomerism, pH, and solvation on the UV/Vis spectrum of sulfapyridine: a joint theoretical and experimental study
Source: J Mol Model. 2026 Sep 12;32(10):360. doi: 10.1007/s00894-026-06933-y (PMC13570832; doi:10.1007/s00894-026-06933-y)
Supplement: Supplementary file 1 [file 894_2026_6933_MOESM1_ESM.zip › Supplementary Information B.pdf]

Supplementary Information B: Unveiling the Effects of Tautomerism, pH and Solvation on the UV/Vis Spectrum of Sulfapyridine: A Joint Theoretical and Experimental Study

José L. F. Santos*[1,2], Lorena C. P. Sales[1], Fernanda L. Souza[1], Gabriel L. C. de Souza[3], Adrian E. Roitberg[2], and Roberto L. A. Haiduke*[1]

[1]Departamento de Química e Física Molecular, Instituto de Química de São Carlos, Universidade de São Paulo, Av. Trab. São Carlense, São Carlos, 13560-970, São Paulo, Brasil

[2]Department of Chemistry, University of Florida, Gainesville, 32611, Florida, USA

[3]Centro de Ciências da Natureza, Universidade Federal de São Carlos, Rod. Lauri Simões de Barros, km 12, Buri, 18290-000, São Paulo, Brasil

*E-mail: jlfelix@usp.br and haiduke@iqsc.usp.br

Table S1: Gibbs energies relative to SPY (ΔG_{rel}) as a function of the number of explicit water molecules (n) for the neutral tautomers obtained at the xTB level within the HCC approach (in kcal.mol^{-1}).

n	ΔG_{rel}^{TAUT}	$\Delta G_{rel}^{TAUT_{HB}}$
0	-2.40	-3.75
1	-1.94	-3.08
2	-2.90	-2.11
3	-4.57	-3.94
4	-5.43	-3.71
5	-4.49	-3.27
6	-4.76	-2.84
7	-5.66	-4.05
8	-4.91	-2.92
9	-3.51	-1.80
10	-6.27	-4.20
11	-6.84	-2.86
12	-6.18	-2.69
13	-5.56	-1.69
14	-5.49	-1.71
15	-8.28	-4.32
16	-7.41	-4.58
17	-10.46	-7.01
18	-8.18	-6.48
19	-11.48	-7.91
20	-9.02	-6.36
21	-12.82	-8.72
22	-12.93	-10.38
23	-13.39	-11.40
24	-11.20	-9.23
25	-10.99	-9.87
26	-10.52	-7.65
27	-9.15	-3.68
28	-8.94	-5.58
29	-10.31	-5.50
30	-9.43	-6.22

Table S2: Gibbs energies relative to SPY(N3)$^+$ (ΔG_{rel}) as a function of the number of explicit water molecules (n) for the protonated tautomers obtained at the xTB level within the HCC approach (in kcal.mol^{-1}).

n	$\Delta G_{rel}^{TAUT(N3)^+}$	$\Delta G_{rel}^{TAUT(N3)^+_{HB}}$	$\Delta G_{rel}^{SPY(N2)^+/TAUT(N1)^+}$	$\Delta G_{rel}^{SPY(N2)^+_{HB}/TAUT(N1)^+_{HB}}$	$\Delta G_{rel}^{SPY(N1)^+}$
0	-4.51	-5.60	-8.53	-11.04	12.98
1	-4.49	-5.01	-8.49	-8.51	12.10
2	-7.97	-9.31	-8.54	-8.21	8.22
3	-6.01	-7.83	-2.84	-3.62	14.45
4	-7.81	-7.03	-2.15	-3.06	11.06
5	-6.20	-5.00	1.08	-0.16	15.33
6	-6.51	-4.14	2.29	1.55	14.82
7	-7.82	-4.15	1.89	2.45	16.00
8	-5.14	-1.15	5.59	5.00	19.38
9	-6.18	-3.16	4.77	4.57	17.91
10	-6.57	-3.14	2.76	3.64	17.49

Table S3: Gibbs energies relative to $SPY(N1)^{-}/TAUT(N2)^{-}$ (ΔG_{rel}) as a function of the number of explicit water molecules (n) for the deprotonated tautomers obtained at xTB level within the HCC approach (in $kcal.mol^{-1}$).

n	$\Delta G_{rel}^{SPY(N3)^-}$	$\Delta G_{rel}^{TAUT(N3)^-}$
0	22.53	22.27
1	23.17	21.44
2	22.85	18.73
3	24.00	17.13
4	25.15	19.83
5	24.77	18.48
6	25.70	19.89
7	27.78	22.60
8	27.15	21.09
9	26.09	21.03
10	27.89	21.77

Table S4: Activation Gibbs energies ($\Delta G^{\ddagger}$), imaginary frequencies of transition states (ω_i), Wigner factors (k_w), rate constants (k_r), half-life times ($t_{1/2}$), and Gibbs energies (ΔG_r) for SPY → TAUT and $SPY(N3)^{+} \rightarrow TAUT(N3)^{+}$ tautomerization reactions with implicit solvation only (SMD) and implicit plus microsolvation (SMD+nW, with n = 1 and 2), as obtained at the CAM-B3LYP/QZ//PBE/TZ level.

	$\Delta G^{\ddagger}$ ($kcal.mol^{-1}$)	ω_i (cm^{-1})	k_w	k_r (s^{-1})	$t_{1/2}$ (s)	ΔG_r ($kcal.mol^{-1}$)
			SPY → TAUT			
SMD	37.15	$1700.5i$	3.81	1.37×10^{-14}	5.06×10^{13}	0.41
SMD+1W	12.53	$1367.8i$	2.82	1.13×10^{4}	6.13×10^{-5}	-0.41
SMD+2W	10.45	$960.7i$	1.90	2.59×10^{5}	6.32×10^{-6}	-1.32
			$SPY(N3)^{+} \rightarrow TAUT(N3)^{+}$			
SMD	35.46	$1699.3i$	3.80	2.40×10^{-13}	2.89×10^{12}	-1.87
SMD+1W	11.83	$1349.1i$	2.77	3.65×10^{4}	1.90×10^{-5}	-2.58
SMD+2W	8.65	$698.7i$	1.47	4.20×10^{6}	1.65×10^{-7}	-2.82

Table S5: Vertical transition energy (VE, in eV) and generalized oscillator strength (GOS) of the first five lowest-lying excited states of TAUT, $TAUT(N3)^{+}$, and $SPY(N1)^{-}/TAUT(N2)^{-}$ forms determined by using CAM-B3LYP/TZ//PBE/TZ calculations in aqueous solution (C-PCM).

Excited State	TAUT	VE	GOS	$TAUT(N3)^{+}$	VE	GOS	$SPY(N1)^{-}/TAUT(N2)^{-}$	VE	GOS
1	NTO-64 → NTO-65 (97%)	4.24	0.268	NTO-64 → NTO-65 (97%)	4.32	0.296	NTO-64 → NTO-65 (96%)	4.54	0.141
2	NTO-64 → NTO-65 (85%)<br>NTO-63 → NTO-66 (12%)	4.77	0.039	NTO-64 → NTO-65 (97%)	5.07	0.216	NTO-64 → NTO-65 (87%)<br>NTO-63 → NTO-66 (12%)	4.77	0.036
3	NTO-64 → NTO-65 (85%)<br>NTO-63 → NTO-66 (12%)	5.00	0.400	NTO-64 → NTO-65 (57%)<br>NTO-63 → NTO-66 (42%)	5.31	0.012	NTO-64 → NTO-65 (87%)<br>NTO-63 → NTO-66 (12%)	4.97	0.419
4	NTO-64 → NTO-65 (96%)<br>NTO-63 → NTO-66 (2%)	5.15	0.054	NTO-64 → NTO-65 (91%)	5.62	0.147	NTO-64 → NTO-65 (43%)<br>NTO-63 → NTO-66 (28%)<br>NTO-62 → NTO-67 (24%)	5.33	0.260
5	NTO-64 → NTO-65 (70%)<br>NTO-63 → NTO-66 (22%)	5.45	0.338	NTO-64 → NTO-65 (77%)<br>NTO-63 → NTO-64 (21%)	5.74	0.008	NTO-64 → NTO-65 (67%)<br>NTO-66 → NTO-66 (18%)<br>NTO-62 → NTO-67 (14%)	5.34	0.109

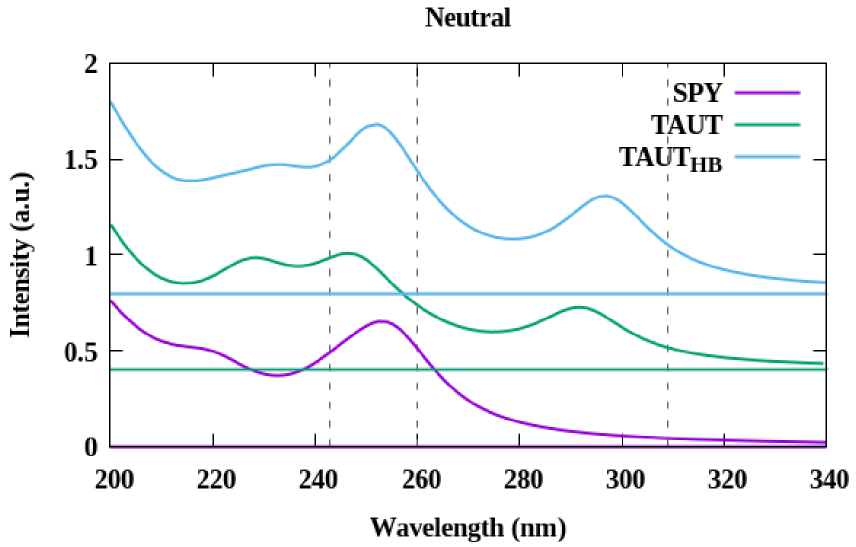

Figure S1: Electronic UV/Vis absorption spectra of neutral tautomers [SPY, TAUT, and TAUT$_{HB}$] determined at the CAM-B3LYP/TZ//PBE/TZ level in aqueous solution (C-PCM). The vertical dashed black lines mark the experimental peak positions.

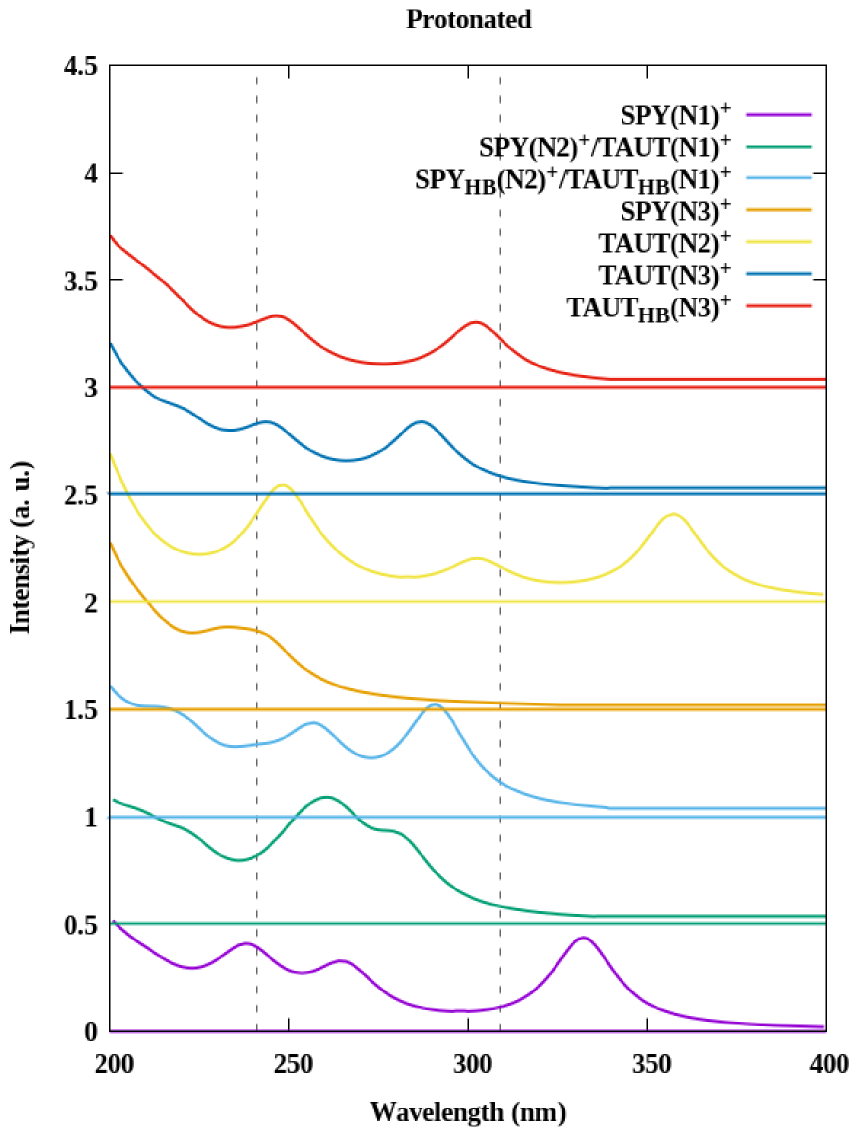

Figure S2: Electronic UV/Vis absorption spectra of protonated tautomers [SPY(N1)$^+$, SPY(N2)$^+$/TAUT(N1)$^+$, SPY$_{HB}$(N2)$^+$/TAUT$_{HB}$(N1)$^+$, SPY(N3)$^+$, TAUT(N2)$^+$, TAUT(N3)$^+$, and TAUT$_{HB}$(N3)$^+$] determined at the CAM-B3LYP/TZ//PBE/TZ level in aqueous solution (C-PCM). The vertical dashed black lines mark the experimental peak positions.

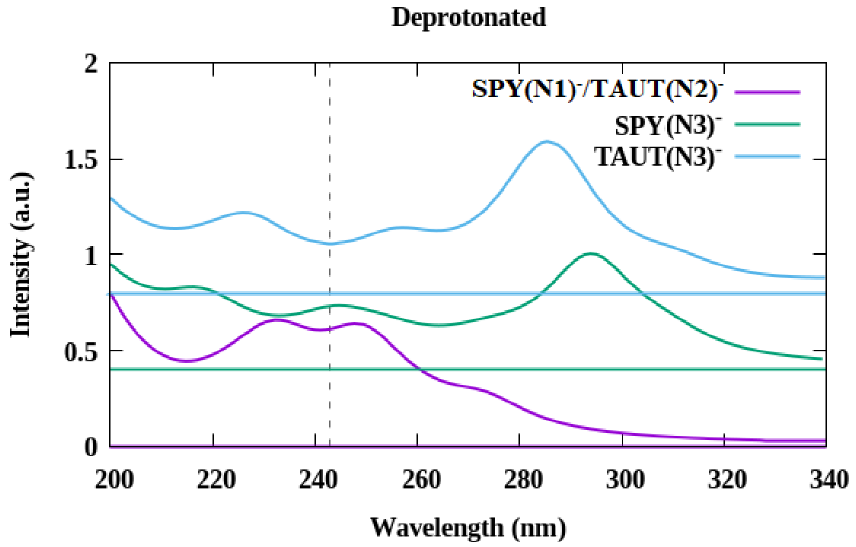

Figure S3: Electronic UV/Vis absorption spectra of deprotonated tautomers [SPY(N1)$^-$/TAUT(N2)$^-$, SPY(N3)$^-$, and TAUT(N3)$^-$ determined at the CAM-B3LYP/TZ//PBE/TZ level in aqueous solution (C-PCM). The vertical dashed black lines mark the experimental peak positions.

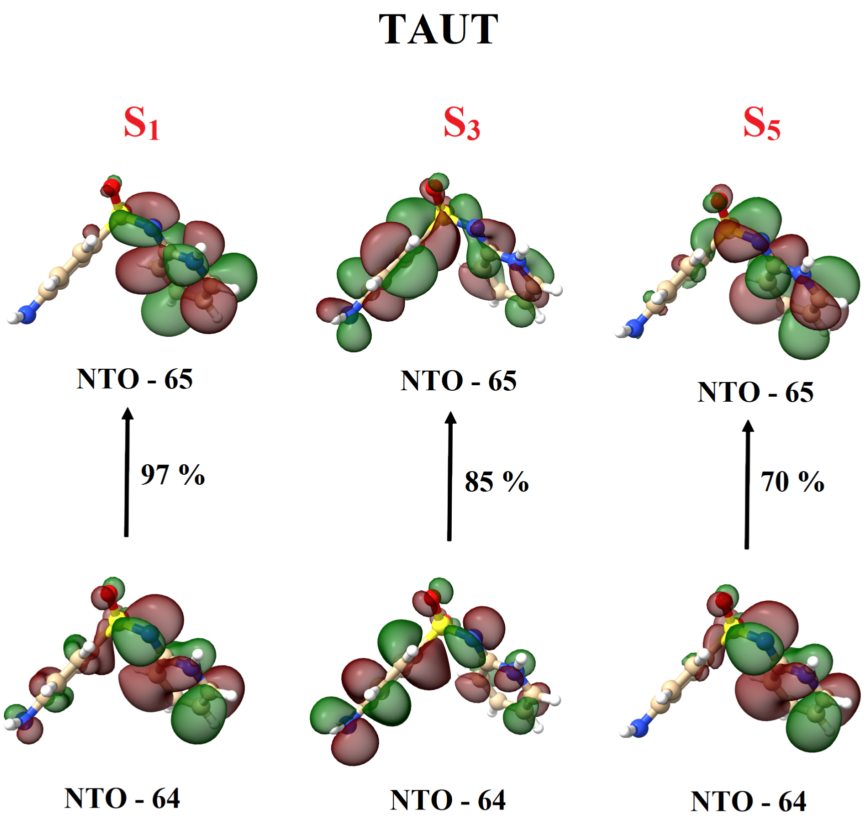

Figure S4: The main NTOs involved in the three most intense electronic absorption transitions of TAUT obtained at the CAM-B3LYP/TZ//PBE/TZ level with C-PCM.

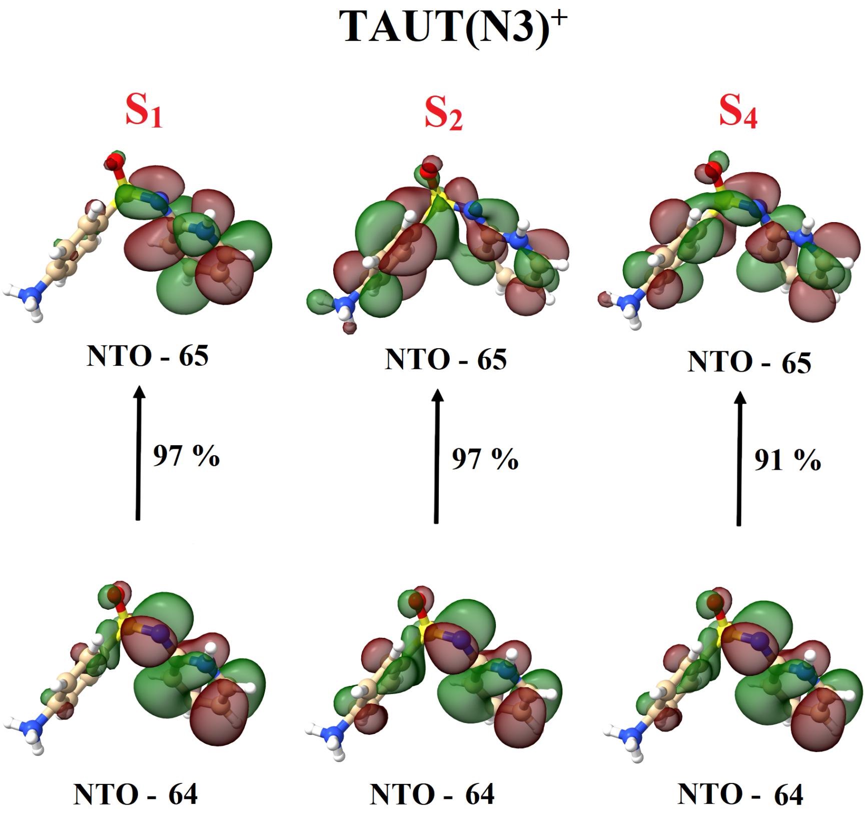

Figure S5: The main NTOs involved in the three most intense electronic absorption transitions of TAUT(N3)$^+$ obtained at the CAM-B3LYP/TZ//PBE/TZ level with C-PCM.

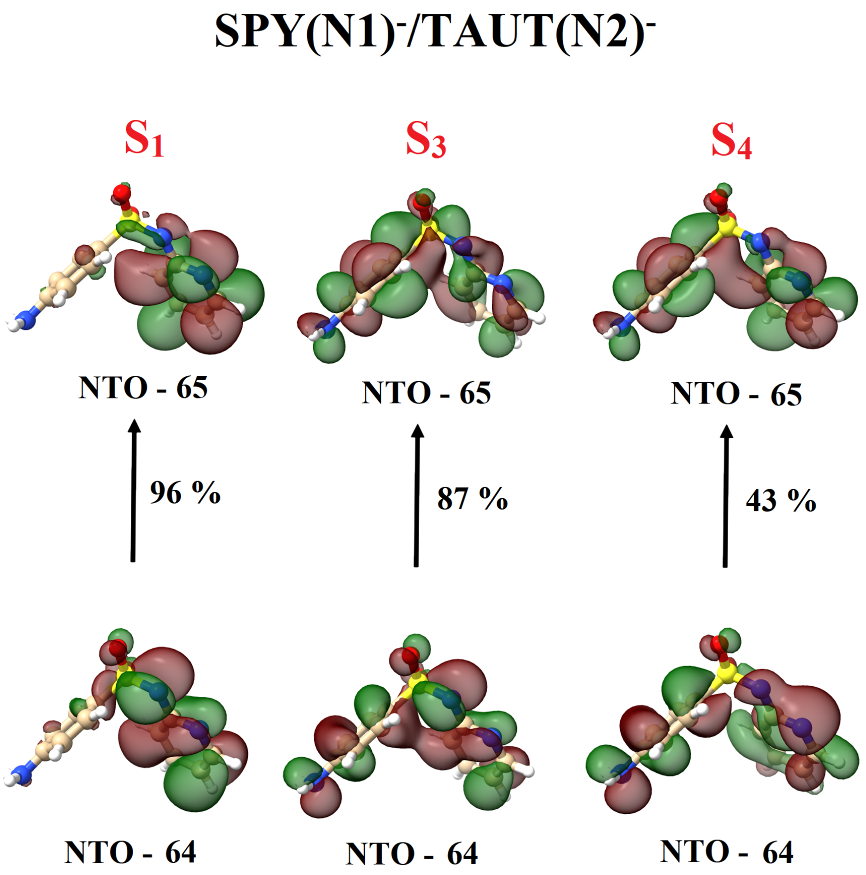

Figure S6: The main NTOs involved in the three most intense electronic absorption transitions of $SPY(N1)^-/TAUT(N2)^-$ obtained at the CAM-B3LYP/TZ//PBE/TZ level with C-PCM.
